# Frequency of polymorphisms and protein expression of cyclin-dependent kinase inhibitor 1A (*CDKN1A*) in central nervous system tumors

**DOI:** 10.1590/S1516-31802009000500008

**Published:** 2010-02-03

**Authors:** Mev Dominguez Valentin, Renata Canalle, Rosane de Paula Queiroz, Luiz Gonzaga Tone

**Affiliations:** I PhD, Genomic and Molecular Laboratory, Research Center of Hospital A. C. Camargo, São Paulo, Brazil.; II PhD. Professor, Biomedicine School, Ministro Reis Velloso Campus, Universidade Federal do Piauí (UFPI), Parnaíba, Piauí, Brazil.; III PhD. Investigator, Childcare and Pediatric Department, Faculdade de Medicina de Ribeirão Preto (FMRP), Universidade de São Paulo (USP), Ribeirão Preto, São Paulo, Brazil.; IV MD, PhD. Professor, Childcare and Pediatric Department. Faculdade de Medicina de Ribeirão Preto (FMRP), Universidade de São Paulo (USP), Ribeirão Preto, São Paulo, Brazil.

**Keywords:** Cyclin-dependent kinase inhibitor p21, Brain neoplasms, Polymorphism, genetic, Polymorphism, restriction fragment length, Blotting, western, Inibidor de quinase dependente de ciclina p21, Neoplasias encefálicas, Polimorfismo genético, Polimorfismo de fragmento de restrição, Western blotting

## Abstract

**CONTEXT AND OBJECTIVE:**

Genetic investigation of central nervous system (CNS) tumors provides valuable information about the genes regulating proliferation, differentiation, angiogenesis, migration and apoptosis in the CNS. The aim of our study was to determine the prevalence of genetic polymorphisms (codon 31 and 3’ untranslated region, 3’UTR) and protein expression of the cyclin-dependent kinase inhibitor 1A *(CDKN1A)* gene in patients with and without CNS tumors.

**DESIGN AND SETTING:**

Analytical cross-sectional study with a control group, at the Molecular Biology Laboratory, Pediatric Oncology Department, Hospital das Clínicas de Ribeirão Preto.

**METHODS:**

41 patients with CNS tumors and a control group of 161 subjects without cancer and paires for sex, age and ethnicity were genotyped using polymerase chain reaction-restriction fragment length polymorphism (PCR-RFLP). Protein analysis was performed on 36 patients with CNS tumors, using the Western Blotting technique.

**RESULTS:**

The frequencies of the heterozygote (*Ser/Arg)* and polymorphic homozygote *(Arg/Arg)* genotypes of codon 31 in the control subjects were 28.0% and 1.2%, respectively. However, the 3’UTR site presented frequencies of 24.2% (C/T) and 0.6% (T/T)*.* These frequencies were not statistically different (P > 0.05) from those seen in the patients with CNS tumors (19.4% and 0.0%, codon 31; 15.8% and 2.6%, 3’UTR site). Regarding the protein expression in ependymomas, 66.67% did not express the protein *CDKN1A*. The results for medulloblastomas and astrocytomas were similar: neither of them expressed the protein (57.14% and 61.54%, respectively).

**CONCLUSION:**

No significant differences in protein expression patterns or polymorphisms of *CDKN1A* in relation to the three types of CNS tumors were observed among Brazilian subjects.

## RESUMO

**CONTEXTO E OBJETIVO:**

A investigação genética dos tumores do sistema nervoso central (SNC) provê valiosa informação sobre os genes que regulam a proliferação, diferenciação, angiogênese, migração e apoptose. O objetivo deste estudo é determinar a prevalência entre os polimorfismos genéticos (códon 31 e da região 3’ não traduzida, 3’UTR) e a expressão protéica do gene inibidor de quinase dependente de ciclina 1A *(CDKN1A)* em pacientes com e sem tumor do SNC.

**TIPO DE ESTUDO E LOCAL:**

Estudo transversal analítico com grupo controle, desenvolvido no Laboratório de Biologia Molecular do Departamento de Oncologia Pediátrica do Hospital das Clínicas de Ribeirão Preto.

**MÉTODOS:**

41 pacientes com tumor do SNC e um grupo controle de 161 indivíduos sem câncer pareados por idade, sexo e etnia foram genotipados mediante uma reação de polimorfismo no comprimento de fragmentos de restrição (RFLP). A análise das proteínas foi realizada em 36 pacientes com tumor de SNC mediante Western Blotting.

**RESULTADOS:**

A frequência do genótipo heterozigoto (*Ser/Arg)* e do homozigoto polimórfico *(Arg/Arg)* do códon 31 nos controles foi 28,0% e 1,2%, respectivamente. Entretanto, o sítio 3’UTR apresentou uma frequência de 24,2% (C/T) e 0,6% (T/T)*.* Estas frequências não são significativamente diferentes (P > 0,05) daquelas observadas no grupo dos pacientes com tumor de SNC (19,4% e 0,0%, códon 31; 15,8% e 2,6%, sítio 3’UTR). Com respeito à expressão protéica, nos ependimomas, 66,67% não expressaram a proteína *CDKN1A*. Estes resultados foram similares entre os meduloblastomas e os astrocitomas, os quais não expressaram a proteína com 57,14% e 61,54%, respectivamente.

**CONCLUSÃO:**

Não foram encontradas diferenças significativas entre o padrão de expressão protéica, polimorfismos de *CDKN1A* e os três tipos de tumores de SNC em indivíduos brasileiros.

## INTRODUCTION

Central nervous system (CNS) tumors are the second most common type of pediatric cancer, exceeded only by leukemia, and they are the most common solid tumor during childhood. Every year, approximately 2,200 subjects younger than 20 years old are diagnosed with CNS tumors in the United States.^1^ The etiology of these tumors is multifactorial and probably varies according to the kind of tumor.

Although the etiology of brain and CNS tumors is largely unknown, genetic investigation of CNS tumors provides valuable information about the genes regulating proliferation, differentiation, angiogenesis, migration and apoptosis in the CNS. Studying the gene expression of this type of neoplasm may identify genes that are candidates for directed gene therapy, in an attempt to increase the chances of cure for CNS tumor subgroups that have an unfavorable prognosis.^2^ One of these genes, cyclin-dependent kinase inhibitor 1A *(CDKN1A),* is a universal inhibitor of cyclin-dependent kinases (CDKs) and plays critical roles in G1/S and G2/M transition regulation. *CDKN1A* may lead to differentiation of normal and transformed cells and suppression of malignant cell growth *in vitro* and *in vivo*.^2^ Moreover, it may lead to apoptosis involving the tumor protein 53 (*TP53)* and retinoblastoma protein (*RB)* signaling pathways, in the same way as in senescence.^3^ Changes in the *CDKN1A* gene and its expression may have an important role in cancer pathogenesis, since its normal function comprises suspension of the cell cycle, terminal differentiation and apoptosis. Different types of neoplasms correlate with *CDKN1A* abnormalities, including cervical neoplasms, colorectal carcinoma, ovary carcinoma, bladder cancer, prostate cancer, hepatomas and others.^4^^,^^5^^,^^6^^,^^7^^,^^8^

Two polymorphisms have been identified and characterized in the *CDKN1A* gene. One of these, the codon 31 transversion (C®A), changes serine into arginine in the protein. The other one is located 20 nucleotides downstream of the termination codon, in the 3’ untranslated region (3’UTR) of exon 3, with a transition of C®T.^9^^,^^10^ These two variants have recently been seen in a significant number of patients with lung, esophageal and breast cancer and sarcomas.

Some studies have analyzed *CDKN1A* gene expression as a possible tool for diagnosis or as a prognosis indicator. Nadal et al.^11^ found that six out of seven laryngeal tumors of grade IV presented low levels of *CDKN1A*, compared with 41 out of 42 tumors of grade I to III, which presented intermediate levels of *CDKN1A*. There is controversy regarding the prognostic value of *CDKN1A* expression in breast tissue. Two groups have associated increased expression with a good prognosis in osteosarcoma,^3^^,^^12^ while other groups have correlated this with a worse prognosis.^13^^,^^14^ The relationships between *CDKN1A* expression levels and the prognostic factors in other types of tumor are similarly contradictory.

Because of the high prevalence of CNS tumors in Brazil (18.3%), and continuing the previous work^15^ that detected the presence of *CDKN1A* gene polymorphisms through sequencing, the goal of this study was to determine the prevalence of *CDKN1A* gene polymorphisms in CNS tumor patients and control individuals. Furthermore, this study sought to analyze associations between these polymorphisms and the risk of developing the disease and its expression, along with the relationship with tumor malignancy grade. As far as we know, this is the first study in Brazil on these polymorphisms among a general population and among CNS tumor patients.

## OBJECTIVE

The aim of this study was to determine the prevalence of genetic polymorphisms (codon 31 and 3’UTR) and protein expression of the cyclin-dependent kinase inhibitor 1A *(CDKN1A)* gene, and to determine the risk of developing CNS-like medulloblastoma (16 cases), astrocytoma (15 cases) and ependymoma (10 cases).

## MATERIAL AND METHODS

### Patients and controls

Tumor tissue stored in the Pediatrics Laboratory tumor bank was used. This tissue came from patients younger than 18 years of age who were treated for medulloblastoma (n = 16), ependymoma (n = 10) and astrocytoma (n = 15) in the Pediatric Oncology and Hematology Department and the Neurosurgery Department of Hospital das Clínicas da Faculdade de Medicina de Ribeirão Preto (HC-FMRP-USP). The use of human tissue was approved by the institution’s Ethics Committee (procedural number 9375/2003).

The criterion used for sample inclusion was the anatomopathological diagnosis, in addition to the availability of samples containing deoxyribonucleic acid (DNA) and proteins in amounts and of quality appropriate for the analysis. The classification of the tumors analyzed was based on the World Health Organization (WHO) nomenclature, 2000.

Genomic DNA samples extracted from peripheral blood lymphocytes were used as controls. These came from the Pediatrics Laboratory controls bank and their use was approved by the Ethics Committee of the participating institution (HC-FMRP-USP) (procedural number 9374/2003). The control group was paired with the patients according to sex, age and ethnicity, and was formed by 97 female and 64 male subjects, with ages ranging from five months to 20 years, without any history of neoplastic or genetic disease. Epidemiological data on the study population were obtained by means of a standard interviewer-administered questionnaire that gathered data on social habits, health problems, family history of cancer and ancestry.

The human subject protocol was approved by the Ethics Committee of HC-FMRP-USP (procedural number 7709/2004), and written informed consent was obtained from all subjects or their parents.

### Polymerase chain reaction

Genomic DNA was extracted using the modified protocol for Trizol reagent-BD (Invitrogen, São Paulo, Brazil). The amplification of the polymerase chain reaction (PCR) was performed using a final volume of 25 ml. The PCR conditions were: 20 mM Tris-hydrochloride (Tris-HCl) (pH 8.4); 50 mM potassium chloride (KCl); 1.5 mM of magnesium chloride (MgCl_2_); 0.1% weight/volume of gelatin; 0.2 mM of deoxyribonucleoside triphosphates (dNTPs); 100 ng of each primer; 1.0 U of Taq DNA polymerase (Invitrogen); and 200 ng of genomic DNA. The primers were designed for the 273 base pairs (bp) of exon 2 of the *CDKN1A* gene (codon 31), with 5’-GGATGTCCGTCAGAACCCAT-3’ (upstream) and 5’- GGTGCCAGGCCGCCTGCCTC - 3’ (downstream). The cycling conditions were: 94 °C for 5 minutes, 34 cycles of 94 °C for 40 seconds, 69 °C for 1 minute and 72 °C for 40 seconds, followed by 10 minutes at 72 °C for the final extension. The primers designed for the amplification of the 300 bp of exon 3 of the *CDKN1A* gene (3’UTR) were 5’-GGGCGGCCAGGGTATGTAC-3’ (upstream) and 5’-CCCAGGGAAGGGTGTCCT G-3’ (downstream). The cycling conditions were 95 °C for 5 minutes, 30 cycles of 95 °C for 30 seconds, 63 °C for 30 seconds and 72 °C for 30 seconds, followed by 10 minutes at 72 °C for the final extension.

### Analysis of restriction fragment length polymorphism

**Figure 1.**
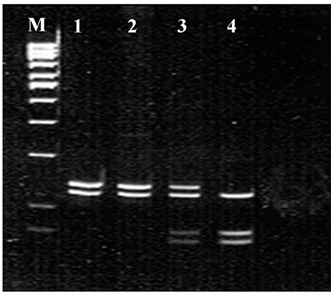
Polymerase chain reaction-restriction fragment length polymorphism (PCR-RFLP) profile showing the polymorphisms in the cyclin-dependent kinase inhibitor 1A (CDKN1A) gene. Analysis of codon 31 of the CDKN1A-BsmAI gene, in which M = molecular weight marker (100 bp); B = blank; lines 1 and 2 = homozygote for wild allele (Ser/Ser); line 3 = heterozygote (Ser/Arg); and line 4 = homozygote for polymorphic allele (Arg/Arg).

**Figure 2.**
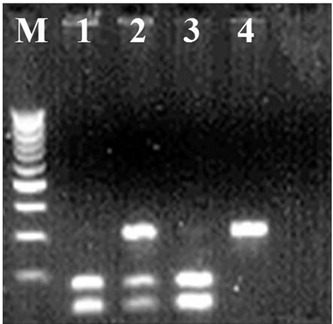
Polymerase chain reaction-restriction fragment length polymorphism (PCR-RFLP) profile showing the polymorphisms in the cyclin-dependent kinase inhibitor 1A (CDKN1A) gene. Polymorphism detection at the 3’ untranslated region (3’UTR), PstI site, in which lines 1 and 3 = homozygote for wild allele (C/C); line 2 = heterozygote (C/T); and line 4 = homozygote for polymorphic allele (T/T).

The 273 bp amplified fragment of exon 2 of the *CDKN1A* gene was produced and digested by the restriction enzyme *BsmA*I (New England Biolabs, Beverey, Massachusetts, United States) using the modified protocol of Mousses et al.^16^ The digestion of the wild type allele (*Ser/Ser*) presented a constant site for restriction enzyme recognition and yielded two bands: one of 142 bp and the other of 131 bp. The homozygote genotype for the polymorphic (*Arg/Arg*) allele was characterized by the presence of a second site for the *BsmA*I enzyme, yielding three bands: 131, 75 and 67 bp; and the heterozygote genotype (*Ser/Arg*) showed four bands: 142, 131, 75 and 67 bp. After digestion, the reaction was analyzed by means of electrophoresis on 10% polyacrylamide gel at 120V (Figure 1). The 300 bp fragment of exon 3 of the *CDKN1A* gene was produced and digested by the *Pst*I enzyme (New England Biolabs) using the modified protocol of Law et al.^17^ The polymorphism C®T gave rise to the loss of the *Pst*I site. *Pst*I digestion of the wild allele, with the *Pst*I site intact, presented two bands: one of 126 bp and the other of 174 bp. After digestion, the reaction was analyzed by means of electrophoresis on 2.0% agarose gel (Invitrogen, São Paulo, Brazil) at 50V (Figure 2).

### Western Blotting

**Figure 3.**
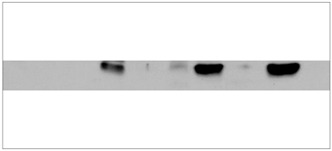
Results from Western Blot experiment showing the cyclin dependent kinase inhibitor 1A (CDKN1A) expression in central nervous system (CNS) tumors. Tu 145, Tu 467 and Tu 449 = no expression of CDKN1A protein; Tu 335, Tu 323, Tu 309, Tu 194, Tu 104 and Tu 100 = expressed CDKN1A protein.

The total protein fraction was obtained by Trizol^®^ reagent (Invitrogen, São Paulo, Brazil). The protein profile was analyzed by means of SDS-PAGE, using the discontinuous system proposed by Laemmli et al.^18^ Mini-gels of dimensions 8 x 7 x 0.01 cm, with acrylamide concentration of 12% in the separation gel and 4% in the stacking gel, were used. The samples were prepared with 50 mg of protein for each 20 ml of sample buffer. The molecular weight marker used was Kaleidoscope Prestained (Bio-Rad, Hercules, California, United States). The proteins were transferred from the polyacrylamide gel into the nitrocellulose membranes through the Bio-Rad system, after their components had previously been immersed in the transfer solution. After running the gel, it was left for 15 minutes in transfer buffer and the transfer was performed at 100V for one hour at 4-6 °C. The primary antibody used was anti-p21 mouse monoclonal (Santa Cruz, Biotechnology, Inc), diluted in 1% Tris-buffered saline Tween (TBST) at a concentration of 1:500, and the secondary antibody was anti-mouse IgG (Amersham Pharmacia Biotech, United States) bound to peroxidase, diluted in 1% TBST at a concentration of 1:2500. The intensity of the bands seen on the X-ray films was quantified using a computerized densitometer (GS 800 Calibrated Densitometer, Bio-Rad). The results were digitized and analyzed (Figure 3) using the reader software (Quantity One quantitation software, Bio-Rad) and their intensity values were expressed in optical density units/square millimeter (OD/mm^2^) and recorded on a chart.

Statistical analysis

Fisher’s exact probability test (Agresti)^19^ was used to analyze the frequency of each genotype studied, in comparison with the total sample of patients and controls. It was also used for comparisons with the different types of tumor, with the aim of possibly correlating the genotypes found with the presence or absence of increased risk of development of CNS tumors. To analyze protein expression, the chi-square test, Fisher’s exact test and the nonparametric Kruskal-Wallis test were used. The significance criterion used was a probability (P) level of less than or equal to 0.05. Odds ratios (OR) and 95% confidence intervals (CI)^20^ were calculated as estimates of risk and degree of association.

## RESULTS

### Genetic analyses of *CDKN1A* in CNS tumors and controls

The frequencies of the C®A polymorphism in codon 31 and C®T polymorphism at the 3’UTR site of the *CDKN1A* gene in patients with CNS tumors and control subjects are shown in Table 1. Among the 36 CNS tumor patients studied for the polymorphism in codon 31, none of them (0.0%) presented the transversion C®A in codon 31. However, out of the 161 controls studied, two subjects (1.2%) showed the transversion leading to the replacement of serine by arginine. The differences in frequency seen between the patients and control group were not statistically significant (P = 1.00), with an odds ratio of 0.78 (CI = 0.04-16.62) (Table 1). For the polymorphism at the 3’UTR site, one patient (2.6%) out of the 38 analyzed showed the C®T transition, and one patient in the control group also showed this (0.6%). There were no statistically significant differences between the patients and the controls (P = 0.37), with an odds ratio of 3.9 (CI = 0.24-64.21) (Table 1).

Analysis of the frequencies of codon 31 genotypes for each CNS tumor type that was evaluated, in comparison with the controls, showed that there were no statistically significant differences between ependymomas and controls (P = 1.00), medulloblastomas and controls (P = 1.00) or astrocytomas and controls (P = 1.00). The same was found regarding polymorphism at the 3’UTR site, and its frequencies were not statistically significant (Table 2).

**Table 1.** Frequency of cyclin-dependent kinase inhibitor 1A (CDKN1A) gene polymorphisms in central nervous system (CNS) tumors and controls

Polymorphisms	Genotype	Number/total (%)		P	OR (95% CI)
		CNS tumors	Controls		
Codon 31	*Ser/Ser*	29/36 (80.6)	114/161 (70.8)	0.31	1.0 (reference)
	*Ser/Arg*	7/36 (19.4)	45/161 (28.0)		0.61 (0.25-1.50)
	*Arg/Arg*	0/36 (0.0)	2/161 (1.2)	1.00	0.78 (0.04-16.62)
3’UTR	*C/C*	31/38 (81.6)	121/161 (75.2)		1.0 (reference)
	*C/T*	6/38 (15.8)	39/161 (24.2)	0.39	0.60 (0.23-1.55)
	*T/T*	1/38 (2.6)	1/161 (0.6)	0.37	3.9 (0.24-64.21)

*Ser/Ser, C/C* = homozygote for wild allele; *Ser/Arg, C/T* = heterozygote; *Arg/Arg, T/T* = homozygote for polymorphic allele; P = values were calculated using Fisher’s exact probability test; OR = odds ratio; CI = confidence interval.

**Table 2.** Frequencies of the cyclin-dependent kinase inhibitor 1A *(CDKN1A)* genotypes in the different types of central nervous system (CNS) tumor and controls

Tumor	Genotype					
	Codon 31			3’UTR		
	*Ser/Ser*	*Ser/Arg*	*Arg/Arg*	*C/C*	*C/T*	*T/T*
Ependymomas	6/9 (66.7)	3/9 (33.3)	0/9 (0.0)	6/10 (60.0)	4/10 (40.0)	0/10 (0.0)
Controls	114/161 (70.8)	45/161 (28.0)	2/161 (1.2)	121/161 (75.2)	39/161 (24.2)	1/161 (0.6)
P		0.72	1.00		0.28	1.00
OR (95% CI)	1.0 (reference)	1.27 (0.30-5.29)	3.5 (0.15-81.26)	1.0 (reference)	2.07 (0.55-7.71)	6.23 (0.23-168.47)
Medulloblastomas	11/14 (78.6)	3/14 (21.4)	0/14 (0.0)	12/14 (85.7)	1/14 (7.15)	1/14 (7.15)
Controls	114/161 (70.8)	45/161 (28.0)	2/161 (1.2)	121/161 (75.2)	39/161 (24.2)	1/161 (0.6)
P		0.76	1.00		0.28	1.00
OR (95% CI)	1.0 (reference)	0.69 (0.18-2.59)	1.99 (0.09-44.08)	1.0 (reference)	2.07 (0.55-7.71)	6.23 (0.23-168.47)
Astrocytomas	12/13 (92.3)	1/13 (7.7)	0/13 (0.0)	13/14 (92.9)	1/14 (7.1)	0/14 (0.0)
Controls	114/161 (70.8)	45/161 (28.0)	2/161 (1.2)	121/161 (75.2)	39/161 (24.2)	1/161 (0.6)
P		0.19	1.00		0.28	1.00
OR (95% CI)	1.0 (reference)	0.21 (0.03-1.67)	1.83 (0.08-40.37)	1.0 (reference)	0.24 (0.03-1.88)	3.00 (0.12-77.40)

*Ser/Ser, C/C* = homozygote for wild allele; *Ser/Arg, C/T* = heterozygote; *Arg/Arg, T/T* = homozygote for polymorphic allele; P = values were calculated using Fisher’s exact probability test; OR = odds ratio; CI = confidence interval.

### Analysis of CDKN1A protein expression in CNS tumors

To determine the clinical relevance of the polymorphisms in codon 31 and at the 3’UTR site, we determined their correlation with CDKN1A protein expression in the CNS tumor samples (Table 3). Out of the 33 tumors evaluated, 39.39% expressed this protein and 60.61% did not (Figure 3). The differences between these two categories were not statistically significant (P = 0.22). The CNS tumors were analyzed in pairs (ependymomas-medulloblastomas, ependymomas-astrocytomas and medulloblastomas-astrocytomas) in order to evaluate their protein expression patterns. It was observed that in ependymomas, 66.67% did not express the CDKN1A protein, whereas the others did so. The results were similar for medulloblastomas and astrocytomas, with 42.86% and 38.46%, respectively. On the other hand, 57.14% and 61.54% did not express this protein. By means of Fisher’s test, it was seen that there was no significant difference (P = 1.00) in expression pattern percentage between the different types of tumor (Table 3). Correlation between the protein expression analyses and polymorphism showed that the two genotypes (*Ser/Ser* and *Ser/Arg*) for polymorphism in codon 31 had similar frequencies for the categories “not expressed” (64.0% and 50.0%) and “expressed” (36.0% and 50.0%). This finding was not statistically significant (P = 0.62), nor was the correlation for polymorphism at the 3’UTR site (P = 0.99) (Table 4).

The polymorphism and protein expression analysis was compared to the degree of malignancy of each tumor. For this, pilocytic astrocytomas (WHO grade I) and ependymomas (WHO grade II) were taken to be low-grade tumors, and anaplastic astrocytomas and ependymomas (WHO grade III) and medulloblastomas (WHO grade IV) were taken to be high-grade tumors. This comparison did not show any significant differences in codon 31 polymorphism (P = 0.99), 3’UTR site polymorphism (p=0.99) or protein pattern (P = 0.86), in relation to the degree of tumor malignancy (Table 5).

**Table 3.** Correlation between protein expression patterns among central nervous system tumors

Tumor	Categories	
	Not expressed	Expressed
Ependymoma	4 (66.67%)	2 (33.33%)
Medulloblastoma	8 (57.14%)	6 (42.86%)
Astrocytoma	8 (61.54%)	5 (38.46%)

**Table 4.** Correlation between polymorphism frequencies and protein expression in central nervous system tumors

Categories	Polymorphisms			
	Codon 31		3’UTR	
	*Ser/Ser*	*Ser/Arg*	*C/C*	*C/T*
Not expressed	16/35 (64.0)	2/4 (50.0)	17/28 (60.7)	1/2 (50.0)
Expressed	9/35 (36.0)	2/4 (50.0)	11/28 (39.3)	1/2 (50.0)
P	0.62		0.99	

*Ser/Ser, C/C* = homozygote for wild allele; *Ser/Arg, C/T* = heterozygote; *Arg/Arg, T/T* = homozygote for polymorphic allele; P = values were calculated using Fisher’s exact probability test.

**Table 5.** Correlation of the polymorphism and protein expression analysis with the clinical behavior of central nervous system tumors

Tumor	Codon 31		3’UTR		WB	
	*Ser/Ser*	*Ser/Arg*	*C/C*	*C/T*	Not expressed	Expressed
LD	14/29 (48.3)	3/7 (42.9)	17/32 (53.1)	3/5 (60.0)	11/16 (68.8)	5/16 (31.3)
HD	15/29 (51.7)	4/7 (57.1)	15/32 (46.9)	2/5 (40.0)	9/17 (52.9)	8/17 (47.1)
P	0.99		0.99		0.86^*^	

3’UTR = 3’ untranslated region; WB = Western Blotting; LD = low-grade tumor; HD = high-grade tumor; Ser/Ser, C/C = homozygote for wild allele; Ser/Arg, C/T = heterozygote; P = values were calculated using Fisher’s exact probability test, *calculated using the chi-square test.

## DISCUSSION

*CDKN1A* plays a central role in suspension of the cell cycle. However, it is possible that gene abnormalities could be responsible for the progression of a number of types of neoplasm.

This was the first Brazilian study to analyze the most frequent *CDKN1A* gene polymorphisms (codon 31 and 3’UTR site) and protein expression relating to the second most frequent category of pediatric cancer (CNS tumors), in comparison with a control population.

Based on these data, we determined the prevalence of genetic polymorphisms of *CDKN1A* in a sample of 10 ependymomas, 16 medulloblastomas and 15 astrocytomas from the northwestern part of the State of São Paulo, compared with 161 control subjects. Our aim was to establish a correlation between these genetic polymorphisms and the risk of developing CNS tumors among children and adolescents. In this study, analyzing the total sample, no difference in the homozygote frequency for the codon 31 polymorphic allele (*Arg/Arg*) was found in CNS tumors (0.0%), in comparison with the controls (1.2%). Regarding the normal homozygote (*Ser/Ser*) and heterozygote (*Ser/Arg*) genotypes, no significant difference was observed between the CNS tumors (80.6% and 19.4%, respectively) and the controls (70.8% and 28.0%, respectively). Our allele frequency for the *Arg* variant (10.0%) was also similar to that observed by Koopmann et al.^21^ in brain tumors (8.5%).

The frequency of the *Arg* allele found in the control population of our study (0.15) was similar to that found by Chedid et al.^22^ in the United States (0.14). It has been suggested that the American and Brazilian populations consist of miscegenation of whites and blacks, since these allele frequencies were found among the values observed by Birgander et al.^23^ in African blacks (0.291) and in European Caucasians (0.039-0.0461). In Brazil, the African influence exerted through the slave population for three centuries, along with the Native Indian and colonizing European populations, could explain these results.^24^ Correlations between each polymorphism and one specific type of tumor did not show any significant differences between ependymomas and controls (P = 0.73), medulloblastomas and controls (P = 0.76) or astrocytomas and controls (P = 0.19), for codon 31. These data did not demonstrate any association between these polymorphisms and the risk of developing cancer. Moreover, similarly to our result, other studies also did not find any association between codon 31 polymorphism and the risk of developing tumors.^5^^,^^9^

The other polymorphism of interest was the 3’UTR site, which plays an important role in mRNA stabilization, cell proliferation and tumor differentiation and suppression.^25^^,^^26^ The prevalence of the homozygote genotype (*C/C*) was found to be 81.6% in the CNS tumors and 75.2% in the controls. The heterozygote genotype (*C/T*) was seen in 15.8% of the CNS tumors and in 24.2% of the controls. The homozygote genotype showed similar percentages for the polymorphic allele (*T/T*) in the CNS tumors (2.6%) and the controls (0.6%).

The frequencies of the polymorphic allele (*T*) were 0.11 in the CNS tumors and 0.13 in the controls. These were higher than the frequency (8%) observed by Shiohara et al.,^27^ and this difference may be related to the ethnic mix of the Brazilian population.^24^

It was observed that all codon 31 and 3’UTR polymorphisms presented linkage disequilibrium. Thus, if a tumor was heterozygotic for codon 31, it was also heterozygotic for the 3’UTR site. Likewise, if it was homozygotic for the wild allele in codon 31, it was also homozygotic for 3’UTR. Facher et al.^10^ found cosegregation of these two polymorphisms in a high percentage of cancer patients, thus suggesting that there was some functional difference in these allele variants that allowed subjects to be more susceptible to certain types of cancer. Polymorphism of the 3’UTR site may be located in a position that is required for rapid degradation of messenger *CDKN1A* and, for this reason, it would prevent transient loss of activity. *CDKN1A* mRNA containing the codon 31 variant could become more stable through association with 3’UTR polymorphism.

Konishi et al.^26^ also suggested that codon 31 polymorphism seemed to be only an innocent ligand and that the 3’UTR site that is tightly bound to codon 31 could be involved in the association between this polymorphism and the risk of tumors. Future experiments, taking this information into account, are required in order to evaluate the possible functional differences that contribute towards carcinogenesis, along with studies involving larger numbers of patients and controls.

*CDKN1A* expression *in vivo*, in human tumors, and its possible role in tumor progression and cell differentiation have been examined by investigators, with conflicting results.^13^ One of the purposes of the present study was to evaluate CDKN1A protein expression in CNS tumors, in order to correlate the expression levels with the development and degree of malignancy of these tumors. Thirty-three CNS tumor samples were analyzed, 6 ependymomas, 13 astrocytomas, and 14 medulloblastomas. We first compared CDKN1A protein expression among the three types of tumor, considering the intensity of the bands obtained through the Western Blotting technique. Analysis of the expression patterns using only two categories (“not expressed” and “expressed”) showed that the differences were not significant, thus indicating that these tumors do not present a uniform pattern of *CDKN1A* expression. It is likely that this non-uniformity of expression pattern occurs because of heterogeneity of the samples and, therefore, we analyzed these two categories in comparison with the different types of tumor. Even though the *CDKN1A* gene has a tumor suppressive function, it has been correlated with increased protein expression in some types of tumor, such as lung carcinoma, hepatocellular carcinoma and head and neck cancer.^13^^,^^14^ In relation to CNS tumors, Jung et al.^28^ found that *CDKN1A* was frequently expressed in greater amounts in astrocytomas, anaplastic astrocytomas and glioblastomas. In addition to investigating the association between polymorphisms and protein expression, we analyzed our results through comparison of these polymorphisms and their expression with the degree of tumor malignancy. The statistical analysis showed that there was no significant difference between the relative clinical behavior of the polymorphisms (P = 0.99 for codon 31 and 3’UTR) and the relative clinical behavior of the protein expression (P = 0.86). These results confirmed the previous analysis, thus showing that there was no correlation between the presence of polymorphisms and the protein expression.

One of the possible explanations for these results is the fact that *CDKN1A* alone is insufficient to stop the cell cycle and inhibit tumor cell growth, considering that mutations or loss of expression of other cell cycle/apoptosis genes such as *PTEN, CDKN2A, MDM2, TP53* and *RB* is also associated with brain tumor etiology. More generally, these pathways are of importance in other cancer types, including breast carcinoma, melanoma and colon carcinoma.^29^ Despite the potential importance of the cell cycle and apoptosis pathways in brain tumor etiology, very little has been published regarding the risk of brain tumors that is associated with the more common gene variants in these pathways, with the exception of the *TP53* gene.^29^ On the other hand, investigation of protein expression levels alone indicates that this is not the active form. It is important to emphasize that the cell cycle regulation pathways are extremely complex and not completely clarified yet.

## CONCLUSIONS

No significant difference was observed between expression patterns and polymorphisms of *CDKN1A* and three kinds of CNS tumors among Brazilian subjects. Therefore, the *CDKN1A* gene could have potential application in gene therapy, because of its role in regulating cell cycle progression or in inducing interruption of the cycle cell.
